# Human amniotic epithelial cell transplantation promotes neurogenesis and ameliorates social deficits in BTBR mice

**DOI:** 10.1186/s13287-019-1267-0

**Published:** 2019-05-31

**Authors:** Ruiyu Zhang, Yulong Cai, Rui Xiao, Hongyu Zhong, Xin Li, Lihe Guo, Haiwei Xu, Xiaotang Fan

**Affiliations:** 1 Department of Developmental Neuropsychology, School of Psychology, Third Military Medical University (Amy Medical University), Chongqing, 400038 China; 2 Institute of Biochemistry and Cell Biology, Shanghai Institutes for Biological Sciences, Chinese Academy of Sciences, Shanghai, 200031 China; 3 Southwest Eye Hospital, Southwest Hospital, Third Military Medical University (Amy Medical University), Chongqing, 400038 China

**Keywords:** hAECs, BTBR, ASD, Hippocampus, Neurogenesis

## Abstract

**Background:**

Autism spectrum disorder (ASD) is a neurodevelopmental disorder characterized by impairments in social interactions and communication and stereotypical patterns of behaviors, interests, or activities. Even with the increased prevalence of ASD, there is no defined standard drug treatment for ASD patients. Currently, stem cells, including human amniotic epithelial cell (hAEC) transplantation, seem to be a promising treatment for ASD, but the effectiveness needs to be verified, and the mechanism has not been clarified.

**Methods:**

We intraventricularly transplanted hAECs into a 2-month-old BTBR T+tf/J (BTBR) mouse model of ASD. Behavior tests were detected 1 month later; hippocampal neurogenesis, neuroprogenitor cell (NPC) pool, and microglia activation were analyzed with immunohistochemistry and immunofluorescence; the levels of pro-inflammatory cytokines, brain-derived neurotrophic factor (BDNF), and TrkB in the hippocampus were determined by real-time PCR or western blotting.

**Results:**

After intraventricular injection of hAECs into adult males, social deficits in BTBR mice were significantly ameliorated. In addition, hAEC transplantation restored the decline of neurogenesis and NPCs in the hippocampus of BTBR mice by expanding the stem cell pool, and the decreased levels of BDNF and TrkB were also rescued in the hippocampus of the hAEC-injected BTBR mice. Meanwhile, the transplantation of hAECs did not induce microglial overactivation or excessive production of pro-inflammatory cytokines in the hippocampus of BTBR mice.

**Conclusions:**

Based on these results, we found that hAEC transplantation ameliorated social deficits and promoted hippocampal neurogenesis in BTBR mice. Our study indicates a promising therapeutic option that could be applied to ASD patients in the future.

## Introduction

Autism spectrum disorder (ASD) is a heterogeneous developmental disability characterized by impairments in social interaction, communication, and repetitive behaviors [1]. The increased prevalence of ASD in recent years represents an important medical and social problem. While the etiology remains elusive, pharmacological interventions mainly target comorbidities rather than the core symptoms, and there is an urgent medical need for new therapeutic options that effectively target the core behavioral deficits related to ASD [2]. The exact pathophysiology of ASD is unclear; studies suggest that many disparate mechanisms are involved. These include neuroimmune processes, GABAergic imbalances, and impaired neurogenesis [3–6].

In humans and other mammals, adult hippocampal neurogenesis is the process by which new granule neurons are produced from the activated radial glia-like neural progenitor cells (NPCs) residing in the subgranular zone (SGZ) of the dentate gyrus (DG) region [7–9]. It is considered that newly generated neurons are highly plastic, and their addition to the brain impacts certain types of higher brain function. It is referred that deficits in hippocampal neurogenesis may be related to the pathology of neurological diseases. Recently, evidence has indicated that deficits in hippocampal neurogenesis contribute to ASD pathogenesis both in humans and in mouse models of ASD [8, 10–12]. Approaches to promote hippocampal neurogenesis also effectively alleviate ASD-like behaviors. It seems that hippocampal neurogenesis could be considered a target for treating ASD.

Stem cell-based regenerative therapies have received much attention for their potential to treat a variety of complicated neurological disorders, such as ASD. It has been indicated that the combined transplantation of human cord blood mononuclear cells and umbilical cord-derived mesenchymal stem cells (MSCs) during childhood improved the ASD rating scale, abnormal behavior checklist scores, and clinical global impression evaluation [13]. Consistent with this study, the transplantation of hematopoietic stem cells (HSCs) from the fetal liver is also beneficial to correct ASD-like symptoms in treating children diagnosed with ASD [14]. Moreover, it has been reported that MSC transplantation corrected stereotypical behaviors and cognitive rigidity to some extent and improved social deficits partially [15]. Human adipose-derived stem cells are also beneficial to correct ASD-like behaviors in valproic acid (VPA)-induced ASD model mice [16]. Nonetheless, several arguments still exist against the wide use of MSCs in clinics, including the probability of tumorigenesis and low cell production from MSC harvesting. Noticeably, human amniotic epithelial cells (hAECs), isolated from the layer closest to the fetus in the term placenta, are referred to as a unique and ideal cell source for cell therapy. hAECs possess pluripotent differentiation ability, low risk of rejection, and tumor generation upon transplantation and are isolated noninvasively in vast abundance without ethical concerns [17]. Furthermore, approximately 1.5 × 10^8^ hAEC cells could be isolated from each amniotic membrane, which is plenty for clinical use without amplification in vitro [17]. Evidence has indicated that hAECs also reduce inflammation and repair established lung injury caused in immune competent mice by bleomycin [18]. The paracrine effect of hAECs could regenerate myocardial tissue and improve cardiac function in a myocardial infarction model [19]. Moreover, hAECs can exert neuroprotection during the acute phase of neuronal injury and promote neuroregeneration in models of CNS disorders [20]. We currently assess the use of hAEC transplantation for ASD treatment.

Animal models are useful in exploring promising treatments in preclinical investigations. The BTBR T+tf/J (BTBR) mouse exhibits several core behavioral deficits of ASD and is thus considered a useful representative model for translational investigations [21, 22]. Meanwhile, inhibited hippocampal neurogenesis was also confirmed in BTBR mice [23]. Here, we detected the efficacy of hAECs in BTBR mice and investigated the cellular mechanism involved. Our findings reveal that hAEC treatment corrected social deficits, which are correlated with the promotion of hippocampal neurogenesis.

## Materials and methods

### Animals

Mice were bred in accordance with the Third Military Medical University guidelines and grouped 3–4 per cage under a 12-h light/dark cycle. All mice were housed in a standard animal facility with free access to water and food. The C57/BL6 mice were provided by the Third Military Medical University, BTBR T+tf/J (BTBR) mice breeding pairs were obtained from the Model Animal Research Center of Nanjing University (Nanjing, China). BTBR male mice (7–8 weeks of age) were randomly allocated into the vehicle and hAEC transplanted groups. The mice were maintained in groups of 3–4/cage and permitted 4 weeks recovery following the operation. All the experimental protocols for this study were approved by the Third Military Medical University and followed the laboratory animal care and use guidelines completely.

### Human amniotic epithelial cells

The present study was approved by the ethics committee of Third Military Medical University. hAECs used in this study were provided by Shanghai iCELL Biotechnology Co., Ltd. The standard culture medium used was Dulbecco’s modified Eagle medium (DMEM) supplemented with 10% fetal bovine serum, 2 mM l-glutamine, 1% nonessential amino acid, 55 μM of 2-mercaptoethanol, 1 mM of sodium pyruvate, 1% antibiotic-antimycotic (all from Gibco, Carlsbad, CA), and 10 ng/mL EGF (Peprotech).

The hAECs grown to a density of 80–90% were trypsinized (0.25% trypsin) and then resuspended in Hank’s balanced salt solution at a concentration of 50,000 cells/μL for transplantation [24].

### Cell transplantation

Under pentobarbital sodium anesthesia, either cells or the excipient (1 μL per injection site, 0.5 μL/min) was bilaterally injected into the lateral ventricles of mice (coordinates from bregma: anterior-posterior, − 0.35 mm; medial-lateral, ± 0.85 mm; dorsal-ventral, − 2.3 mm). After the completion of the injections, the needle was left in situ for 5 min before being slowly removed. To suppress the possible immune response, mice received 15 mg/kg cyclosporine (Hangzhou Zhongmei Huadong Pharmaceutical Co., Ltd.) intraperitoneal (i.p.) injection for 3 days after transplantation. Then, oral cyclosporine was used in drinking water (15 mg/kg) for the entire experimental period before the mice were killed [16, 25].

### Behavioral tests

One month after transplantation, behavioral tests were conducted between 10:00 and 17:00. At least 30 min before testing, the animals were transferred from their colony to a sound-attenuating behavioral testing room. One test was done per day until the assessments were completed. Seventy percent of ethanol was used to clean the apparatus after each trial.

After behavioral tests, to evaluate cell proliferation in the hippocampal DG, animals were given bromodeoxyuridine (BrdU; Sigma-Aldrich) at a dose of 100 mg/kg (i.p.) twice daily for 3 days before being killed.

### Three-chamber social test

The sociability was tested in a three-chambered apparatus (40 cm × 60 cm × 22 cm). The mice were allowed to access into each chamber through the retractable doorways within the two dividing walls. An overhead camera recorded the behavior for subsequent analyzing. Briefly, the test was composed of a habituation session and a social approach session. During the habituation session, mice were allowed to move in all chambers freely for 10 min. During the social approach session, a novel mouse (S) and a novel object (O) were placed in the two sides of the chamber. The subject mouse was allowed to explore three chambers for 10 min. The novel strain and sex-matched mouse was aged 11–12 weeks and acclimatized to the plastic cage for 30 min on the previous day. Lights were set at 40 lx during the sociability detection. The time explored in each chamber and sniffing during each test session was analyzed using Noldus Observer software [26, 27]. The preference index was calculated, which refers to the numerical time difference between chambers or sniffings (S versus O) divided by total time in both chambers and sniffings.

### Self-grooming

Each mouse was introduced individually into a clean, standard home cage. The repetitive self-grooming behaviors of the mice were video recorded for 20 min, illuminated at 40 lx. The second 10 min was considered a testing period and manually scored [28, 29].

### Marble burying

Each mouse was placed in a clean home cage (27 cm × 16.5 cm × 12.5 cm) containing 20 marbles (1.5 cm in diameter) evenly arranged in a 4 × 5 grid throughout the bedding to a depth of 2–3 cm. The number of buried marbles was scored (30 min test duration). Marbles covered by bedding > 50% were defined as “buried.” Testing was conducted under low illumination of 15 lx [30].

### Open-field test

General locomotor activity was measured in an open-field apparatus [31]. The apparatus consisted of gray plexiglass sides and floors, approximately 40 cm × 40 cm × 30 cm. The test mouse was initially placed in the center of the arena and video recorded the movements for 30 min. Total distance traveled and time spent in the center were acquired and analyzed using Ethovision 11.0 (Noldus).

### Elevated plus maze

The elevated plus maze test was performed to detect the anxiety-like behavior [8, 27]. The apparatus consisted of two open arms (30 cm × 6 cm × 15 cm) and two closed arms (30 cm × 6 cm × 15 cm) radiating from a central area (6 cm × 6 cm). Briefly, each mouse was initially placed at the center of the maze facing an open arm and recorded for 10 min. An arm entry was determined by four paws of the mouse within an arm. Lights were set at ~ 300 lx during the test. The percentage of time spent in the open arms and the total open arm entries were analyzed.

### Light-dark test

The light-dark test was also performed to assess the anxiety levels in mice [31]. The mouse was initially placed in the light chamber (~ 400 lx) and recorded for 10 min. Time spent in the dark chamber and the total transitions between chambers were analyzed using Noldus Observer software.

### Immunohistochemistry and immunofluorescence

According to our previous study [32], the anesthetized mice were perfused by cardiac infusion of saline followed by 4% paraformaldehyde (PFA). The brains were post-fixed in 4% PFA and then placed in 30% sucrose in PFA for 24 h. The brain tissues were cut coronally into 30 μm slices on a cryostat at − 20 °C and maintained at − 20 °C in a cryoprotectant solution. Briefly, the sections were incubated with primary antibodies for 12 h at 4 °C: anti-Iba1 (1:1000, Wako, CA, USA), anti-DCX (1:100, Santa Cruz Biotechnology, USA), anti-Sox2 (1:500, Abcam, Cambridge, UK), anti-Prox1 (1:500, Covance, USA), anti-glial fibrillary acidic protein (GFAP) (1:500, Santa Cruz Biotechnology, USA), and anti-Nestin (1:400, BD Pharmingen, USA). For BrdU immunostaining, the sections were pretreated with 2 N HCl prior to incubation with mouse anti-BrdU (1:200, BD Biosciences). For immunohistochemistry, the sections were further processed using the avidin-biotin-peroxidase method and imaged using a Zeiss microscope (Oberkochen, Germany). For immunofluorescence, the sections were incubated with Cy3- or 488-conjugated secondary antibodies (both at 1:500, Jackson ImmunoResearch) for 3 h at room temperature, followed by counterstaining with 4′,6-diamidino-2-phenylindole (DAPI, Beyotime, China). The images of immunofluorescence were scanned on a confocal laser scanning microscope (Leica TCS-SP2; Heidelberg, Germany) and analyzed with Leica imaging software.

### Real-time PCR

The hippocampi were rapidly removed from the mouse brain after completion of the behavioral tests; total RNA was isolated from the hippocampus using Trizol reagent (Invitrogen, USA). Subsequently, reverse transcription was performed to prepare cDNA, followed by real-time PCR using primers designed against the IL-1β, IL-6, IL-10, NF-κB, TNF, DCX, Nestin, Sox2_,_ Prox1, BDNF, TrkB, and GAPDH genes. Assays were performed using primers in a final volume of 10 μL containing 0.5 μL cDNA template, 2 × TB Green™ Priemix ex taq™ ІІ (Takara), 0.8 μL of 10μΜ/μL each primer, and nuclease-free water. Cycling conditions were 15 min at 95 °C, followed by 40 cycles at 95 °C for 15 s, 60 °C for 20 s, and 72 °C for 20 s in a CFX CONNETCT™ (BIO-RAD). After all the amplification cycles, a melting curve was run. Each sample was tested in triplicate. GAPDH served as an internal standard. And the changes were calculated using the 2^−ΔΔCt^ method. The primer sequences used are as follows: IL-1β forward primer 5′-GGCAACTGTTCCTGAACTCAACTG-3′, reverse primer 5′-CCATTGAGGTGGAGAGCTTTCAGC-3′; IL-6 forward primer 5′-CTGCAAGAGACTTCCATCCAG-3′, reverse primer 5′-AGTGGTATAGACAGGT CTGTTGG-3′; IL-10 forward primer 5′-CTTACTGACTGGCATGAGGATCA-3′, reverse primer 5′-GCAGCTCTAGGAGCATGTGG-3′; NF-κB forward primer 5′-ATGGCAGACGATGATCCCTAC-3′, reverse primer 5′-CGGAATCGAAATCC CCTCTGTT-3′; TNF forward primer 5′-ACGTGGAACTGGCAGAAGAG-3′, reverse primer 5′-GGTCTGGGCCATAGAACTGA-3′; DCX forward primer 5′-TTTGGACATTTTGACGAACGAGA-3′, reverse primer 5′-GTGGGCACTATGAGTGGGAC-3′; Nestin forward primer 5′-CCCCTTGCCTAATACCCTTGA-3′, reverse primer 5′-GCCTCAGACATAGGTGGGATG-3′; Sox2 forward primer 5′-GCGGACTGGAAACTTTTGTCC-3′, reverse primer 5′-GGGAAGCGTGTACTTATCC-3′; Prox1 forward primer 5′-AGAAGGGTTGACATTGGAGTGA-3′, reverse primer 5′-TGCGTGTTGCACCACAGAATA-3′; BDNF forward primer 5′-TCATACTTCGGTTGCATGAAGG-3′, reverse primer 5′-ACACCTGGGTAGGCCAAGTT-3′; TrkB forward primer 5′-GTTGACCGGAGAACATCACG-3′, reverse primer 5′-ACTTTAAGCCGGAA; and GAPDH forward primer 5′-AGGTCGGTGTGAACGGATTTG-3′, reverse primer 5′-TGTAGACCATGTAGTTGAGGTCA-3′.

### Western blot

The hippocampi were isolated and homogenized in ice-cold RIPA lysis buffer (Beyotime, Shanghai, China). The protein concentration was measured using a BCA kit (Beyotime Institute of Biotechnology, Shanghai, China). A protein (30 μg) from each sample was subjected to electrophoresis on 8% SDS-polyacrylamide gel (120 min at 80 V) and then transferred onto a PVDF membrane under constant electricity of 220 mA for 90 min. After blocking, the PVDF membranes were incubated with anti-BDNF (1:1000, Abcam, Cambridge, UK), anti-TrkB (1:1000, Cell Signaling Technologies), and anti-GAPDH (1:2000, Santa Cruz Biotechnology) overnight at 4 °C, followed by incubation for 1 h at room temperature with peroxidase-conjugated secondary antibodies (1:2000,Santa Cruz Biotechnology). The immunoblots were visualized by the enhanced chemiluminescence method (Amersham, Piscataway, NJ, USA). The relative intensities were quantified in relation to GAPDH and then normalized to the control values. Each experiment was triplicated. Three animals in each group were used in the statistical analysis.

### Cell counting, unbiased stereology, and quantification of microglial activation state

Radial glial cells (RGCs) underwent immunofluorescent double staining with Sox2 and GFAP; the processes labeled by GFAP usually extend into the molecular layer. The number of Iba1^+^, Nestin^+^, Sox2^+^, Sox2^+^/GFAP^+^, and DCX^+^ in the SGZ and Prox1^+^ in the GCL of five matched sections in each mouse was counted, and the average count of each section was calculated for each mouse. Four mice in each group were used for the analysis.

Stereological cell counting was used to quantify the total number of BrdU^+^ cells in the DG (GCL plus SGZ) [32]. Briefly, every tenth section (30 μm thickness) throughout the hippocampus was selected. The total sum of the BrdU^+^ cells traced was multiplied by the count in each section and series number to give the total values in the DG. Four mice in each group were used for analysis.

The activation state of microglia was categorized from 0 (lowest activation) to 3 (highest activation) based on the following criteria [33]. The process morphology was scored as 0 (> 15 thin processes with multiple branches), 1 (5–15 thick processes with branches), 2 (1–5 thick processes with few branches), and 3 (no clear processes). The percentage of microglial cells was counted in each category.

### Statistical analysis

Paired *t* tests were used to analyze the time spent in the chamber and sniffing within each group in the three-chamber social test. Mauchly’s test was used to evaluate the center time and total distance in the open-field test. The rest of the results were analyzed using one-way ANOVA followed by Tukey’s least significant difference post hoc test for multiple comparisons. Statistical analysis was performed using SPSS 24.0 software (SPSS Inc., Chicago, IL, USA). Data are presented as the mean ± SEM. A *P* value of less than 0.05 was considered statistically significant.

## Results

### hAEC injection ameliorated social deficits in the three-chambered social approach task of BTBR mice, but not the repetitive behavior

**Fig. 1:**
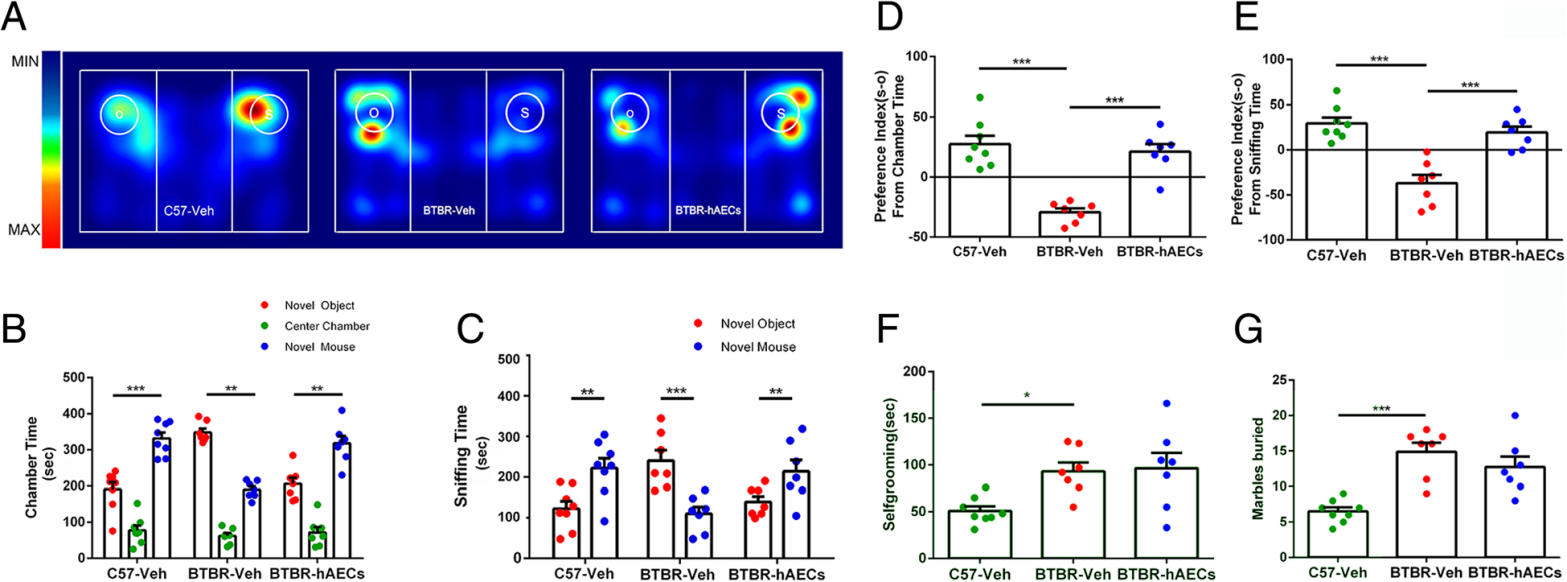
hAECs reversed social deficits in the three-chambered social test in BTBR mice but did not reduce repetitive behaviors. **a** Representative heat maps of resting time of BTBR and C57 mice in the sociability chamber. “O” and “S” represent object and mouse, respectively. **b** C57 mice displayed normal sociability on the chamber time parameter and spent more time in the chamber with the novel mouse compared to the novel object after vehicle treatment. BTBR mice exhibited their lacks of sociability characteristic on the chamber time parameter. However, these social deficits were reversed in BTBR mice with hAEC injection. **c** The C57 mice treated with vehicle exhibited characteristic sociability on the directed sniffing parameter; BTBR mice after vehicle treatment displayed more interest in the novel object than the novel mouse, but BTBR mice following hAEC injection were more inclined to the novel mouse. **d** BTBR mice with vehicle injection displayed a lower reference index (S − O/total) compared to C57 mice in chamber time. After hAEC injection, the preference index (S − O/total) was increased in BTBR mice. **e** BTBR mice showed a lower preference index (S − O/total) in sniffing time compared to C57 mice, and hAEC injection improved the impaired sociability by increasing the preference index (S − O/total) in sniffing time. **f** BTBR mice displayed normally high levels of self-grooming compared to C57 mice; hAECs could not reduce self-grooming for BTBR mice. **g** BTBR mice buried more marbles than C57 mice, and BTBR mice treated with vehicle or hAECs showed no difference in marble burying. The data are presented as the mean ± SEM (*n* = 7–8). *P < 0.05, **P < 0.01, ***P < 0.001

Sociability was defined as a preference for the novel mouse over the novel object. The C57 mice exhibited normal sociability (Fig. 1a, b, *P* < 0.001). The vehicle-treated BTBR mice spent significantly more time in the chamber with the novel object than with the novel mouse (Fig. 1a, b, *P* < 0.01). From this, we can infer that BTBR mice with vehicle treatment exhibited typical deficits in sociability. However, these decreased social explorative activities in BTBR mice could be reversed after hAEC injection (Fig. 1a, b, *P* < 0.01). Moreover, vehicle-treated BTBR mice displayed a lower preference index (S − O/total) in chamber time (Fig. 1d, *P* < 0.001) compared to C57 mice, and BTBR mice displayed a higher preference index (S − O/total) after hAEC injection compared to the vehicle group (Fig. 1d, *P* < 0.001).

We also use sniffing time as a more sensitive and direct assay to evaluate sociability. The C57 mice exhibited normal sociability (Fig. 1c, *P* < 0.01). However, BTBR mice with vehicle administration exhibited significantly more sniffing of the novel object over the novel mouse (Fig. 1c, *P* < 0.001). These social deficits were also reversed in the hAECs group (Fig. 1c, *P* < 0.01). In addition, BTBR mice injected with hAECs showed a higher preference index (S − O/total) compared to the vehicle group (Fig. 1f, *P* < 0.001).

Next, we further investigate whether hAEC treatment affects stereotyped repetitive behaviors using marble burying and self-grooming. Vehicle-treated BTBR mice displayed higher self-grooming time (Fig. 1f, *P* < 0.05) and buried more marbles (Fig. 1g, *P* < 0.001) compared to C57 mice treated with the vehicle. Observational analysis determined that hAEC treatment did not reduce repetitive behaviors since there was no significant difference between hAEC-treated BTBR mice and vehicle-treated BTBR mice in the self-grooming test (Fig. 1f, NS) or the marble burying task (Fig. 1g, NS).

### hAECs had no effects on physical activity or anxiety behavior of BTBR mice

**Fig. 2:**
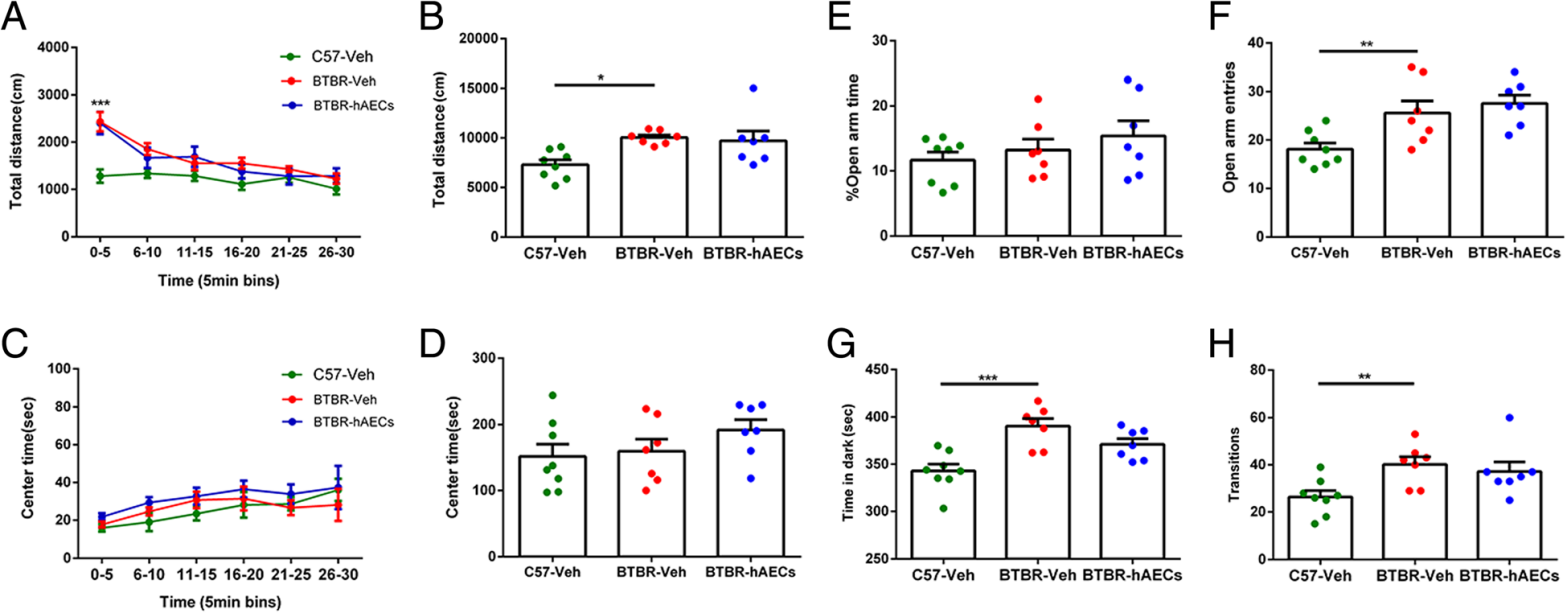
hAEC treatment did not affect the general exploratory locomotion and anxiety-like behavior in BTBR mice. **a**–**d** Behavioral performance in the open field. **a**, **b** BTBR mice with vehicle treatment moved more than C57 mice in total distance and in the first 5 min. **c**, **d** There was no significant difference in the three groups in the center time. **e**, **f** Behavioral performance in the elevated plus maze. Vehicle-treated BTBR mice exhibited more open arm entries than C57 mice, and hAEC treatment did not significantly affect the percentage of time spent in the open arm or the number of open arm entries. **g** Vehicle-treated BTBR mice spent more time in the dark box than C57 mice, and hAEC injection did not reduce the time BTBR mice spent in the dark box. **h** Observational analysis showed no difference in the transitions among the groups. The data are presented as the mean ± SEM (*n* = 7–8). *P < 0.05, **P < 0.01, ***P < 0.001

The open-field test was carried out in the three groups to detect whether hAEC treatment altered general activity, which could produce confounding effects on the sociability test. The total distance over 30 min in 5-min bins was measured, and BTBR mice moved more than C57 mice in the first 5 min (Fig. 2a, *P* < 0.001). Additionally, BTBR mice moved more than C57 mice in the whole 30 min (Fig. 2b, *P* < 0.05), but hAECs did not change the total distance in BTBR mice (Fig. 2b, NS). Observational analysis determined that hAECs had no effect on anxiety. The center time over 30 min was measured, and no significant difference was detected among the three groups (Fig. 2c, f (2,19) = 1.414, NS; Fig. 2d, NS). We also illustrated the anxious effects of hAECs assessed using the standard elevated plus maze. hAECs did not cause any significant anxious effects because they did not alter the percentage of the time in open arms (Fig. 2e, NS) or the total number of open arm entries (Fig. 2f, NS). Furthermore, hAEC treatment did not change the cumulative time spent in the dark chamber (Fig. 2g, NS) and transitions between the light and dark compartments in BTBR mice (Fig. 2h, NS). These data from three paradigms indicate that hAEC administration did not induce anxiety-like behavior in BTBR mice.

### hAECs had no effects on hippocampal microglia of BTBR mice

**Fig. 3:**
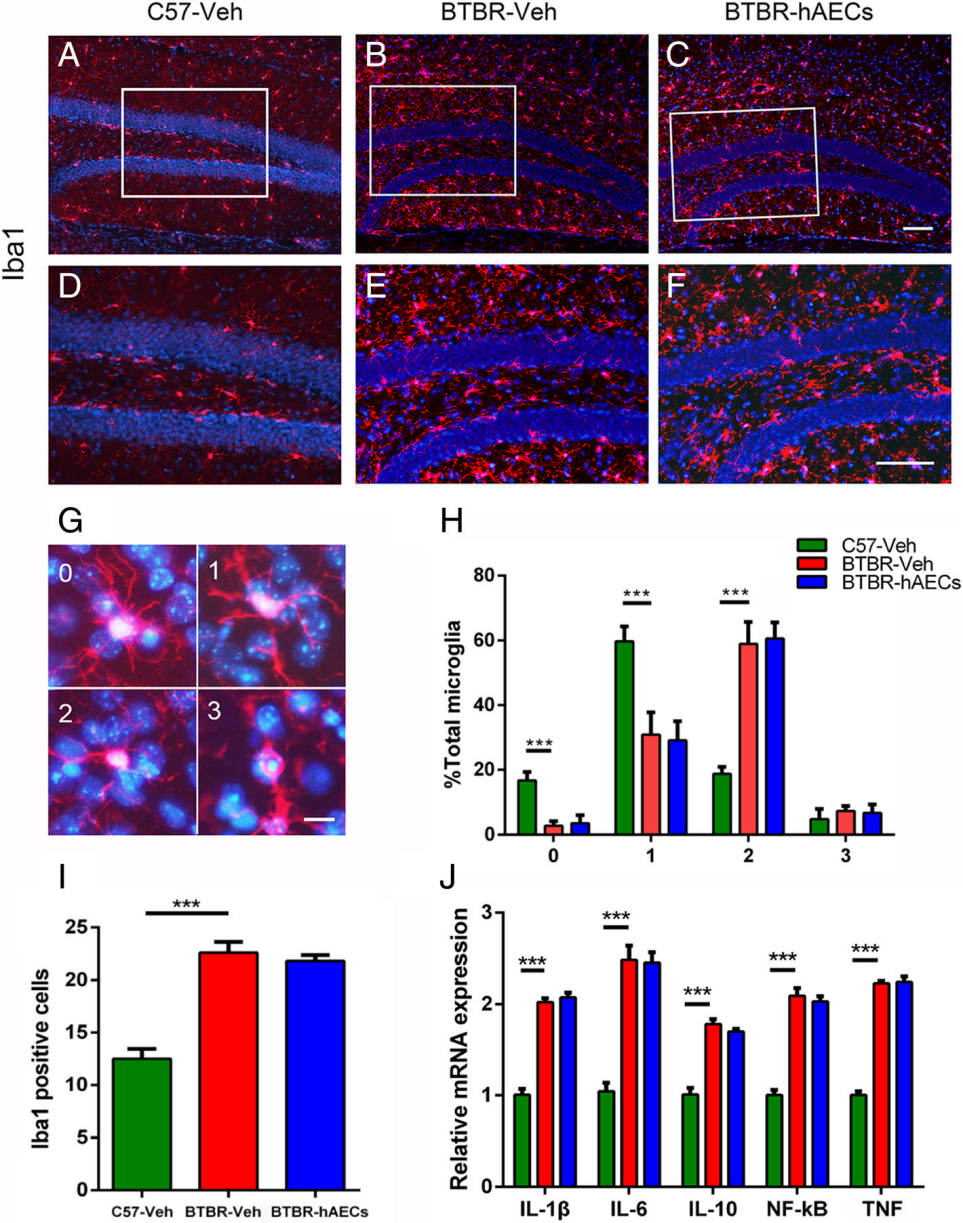
hAEC treatment did not alter microglia activation and inflammatory factors in the hippocampus of BTBR mice. **a**–**c** Microglia cells labeled by Iba1 in the DGs. **d**–**f** Magnified views of the boxed areas in **a**–**c**. **g** Microglia cells labeled by Iba1 of each category. **h** Quantitative analysis of the percentage of Iba1^+^ cells of each category. **i** Quantitative analysis of the number of Iba1^+^ cells in the SGZ. **j** In the hippocampus, the relative mRNA expression levels of IL-1β, IL-6, IL-10, TNF, and NF-κB, which are inflammatory cytokines, were expressed in high levels in BTBR mice with vehicle treatment compared to C57 mice; these expression levels were the same in the vehicle-treated and hAEC-treated BTBR mice. The data are presented as the mean ± SEM (*n* = 4 for immunohistochemistry, *n* = 3–4 for RT-qPCR). Scale bar in **c** = 50 μm and applies to **a**–**c**; in **f** = 50 μm and applies to **d**–**f**; in **g** = 20 μm

Inflammation is involved in the development of ASD. Therefore, the quantification of microglia was examined in this study. We found that BTBR mice with vehicle treatment had more Iba1^+^ microglia in the hippocampal SGZ compared to C57 mice (Fig. 3a, b, d, e, i, *P* < 0.001), and there was no significant difference in the number of Iba1^+^ microglia in the SGZ between the BTBR mice treated with vehicle and hAECs (Fig. 3b–f, i, NS). We further categorized the microglial activation state by determining a morphological score with grid analysis in the hippocampus. We confirmed that more microglia were activated in the hippocampus of BTBR mice compared with that in the C57 mice, which could not be altered by hAEC treatment (Fig. 3g, h). Pro-inflammatory cytokines were also analyzed in the hippocampus of the BTBR mice. As shown in Fig. 3h, the mRNA levels of IL-1β, IL-6, IL-10, NF-κB, and TNF in the hippocampus of BTBR mice treated with vehicle were higher than in C57 mice (Fig. 3j, *P* < 0.001) and were not altered by hAEC treatment (Fig. 3j, NS).

### hAEC treatment increased hippocampal neurogenesis in BTBR mice

**Fig. 4:**
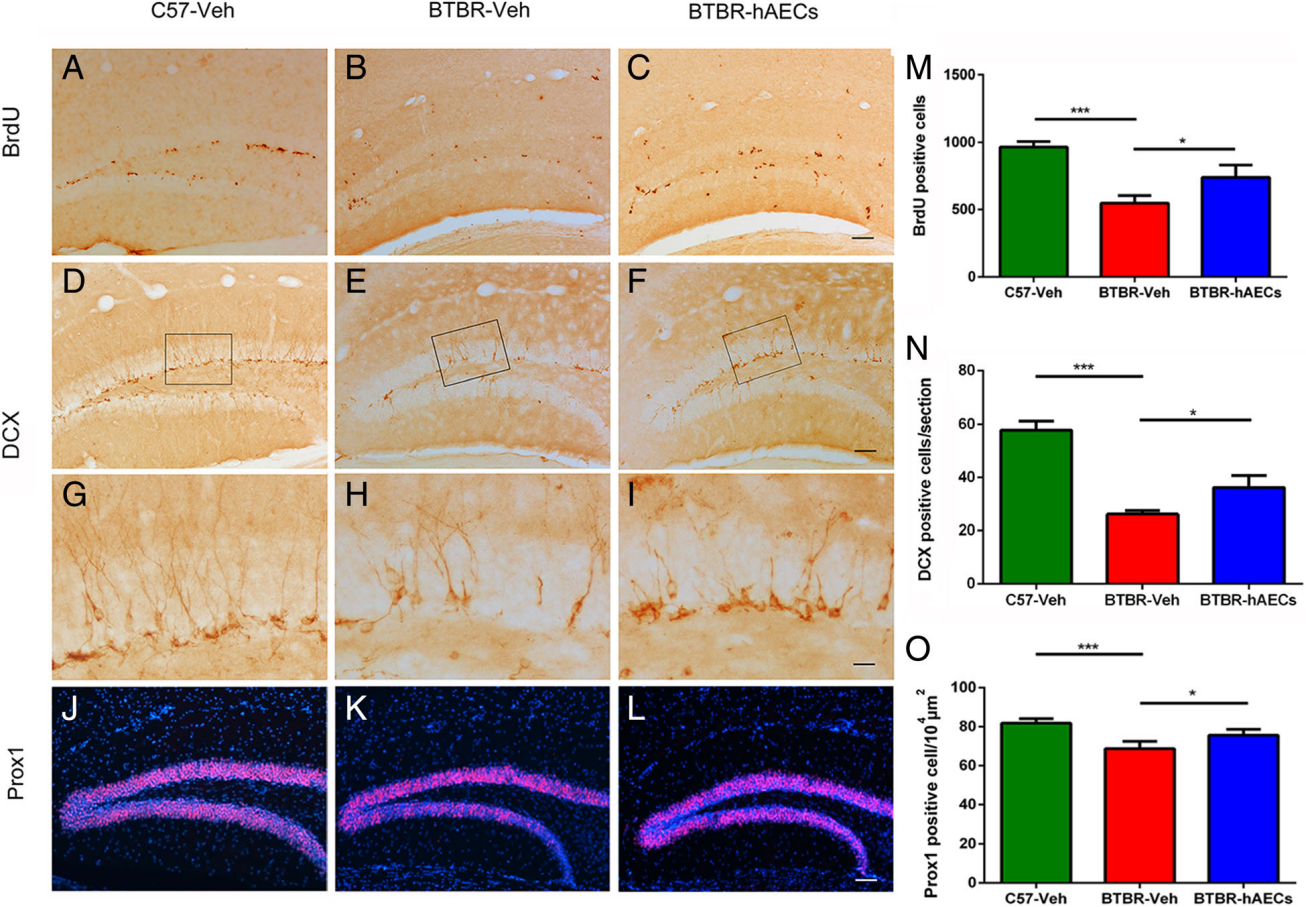
hAEC treatment increased BrdU- and DCX-positive cells in the DG of BTBR mice. **a**–**c** Representative images of BrdU-labeled sections of the DG for each group. **d**–**f** Representative images of DCX-labeled sections of the SGZ for each group. **g**–**i** The images are the magnified views of the boxed areas in **d**–**f**. **j**–**l** Representative images of Prox1-labeled sections of the SGZ for each group. **m** Quantitative analysis of the number of BrdU-labeled cells in the hippocampal DG. C57 mice exhibited more BrdU^+^ cells in the DG compared to BTBR mice with vehicle treatment. After hAEC treatment, the number of BrdU^+^ cells increased in the DG of the BTBR mice. **n** Quantitative analysis of the number of DCX-labeled cells in the hippocampal SGZ. C57 mice also exhibited more DCX^+^ cells in the SGZ compared to vehicle-treated BTBR mice, and the number of DCX^+^ cells was also increased after hAEC treatment in BTBR mice. **o** Quantitative analysis of the density of Prox1-labeled cells in the DG. BTBR mice exhibited a lower density of Prox1^+^ cells in the DG compared to C57 mice, and the number of Prox1^+^ cells was also increased after hAEC treatment in BTBR mice. The data are presented as the mean ± SEM (*n* = 4). *P < 0.05, **P < 0.01, ***P < 0.001. The scale bar in **c** = 50 μm and applies to **a**–**c**; in **f** = 50 μm and applies to **d**–**f**; and in **i** = 20 μm and applies to **g**–**i**; and in **l** = 50 μm and applies to **j**–**l**

It has been highlighted that neurogenesis deficits are involved in ASD pathogenesis. Stimulators of neurogenesis could produce therapeutic benefits in BTBR mice. To explore whether hAEC treatment affects hippocampal neurogenesis, brain tissue was immunostained with BrdU, a marker of cell proliferation. Stereological analysis revealed that compared to the C57 mice, vehicle-treated BTBR mice exhibited fewer BrdU-positive cells in the DG (Fig. 4a, b, j, *P* < 0.001). In addition, hAEC treatment in BTBR mice significantly increased the number of BrdU-positive cells compared to the vehicle-treated group (Fig. 4b, c, j, *P* < 0.05). DCX, an early neuronal marker, was further assayed in the DG of the hippocampus. BTBR mice treated with vehicle exhibited fewer DCX-positive cells in the SGZ compared to the C57 mice (Fig. 4d, e, g, h, k, *P* < 0.001). There was also a 38.2% increase in cells expressing DCX in the SGZ of the hAEC-treated mice (Fig. 4e–i, k, *P* < 0.05). Prox1, a marker of intermediate progenitor cells, was further assayed in the DG of the hippocampus [34]. Statistical analysis revealed that vehicle-treated BTBR mice exhibited a lower density of Prox1-positive cells in the GCL (Fig. 4j, k, o, *P* < 0.001). hAEC treatment in BTBR mice significantly increased the density of Prox1-positive cells compared to the vehicle-treated group (Fig. 4k, l, o, *P* < 0.05).

### hAEC injection increased the NPC pool in the DG of BTBR mice

**Fig. 5:**
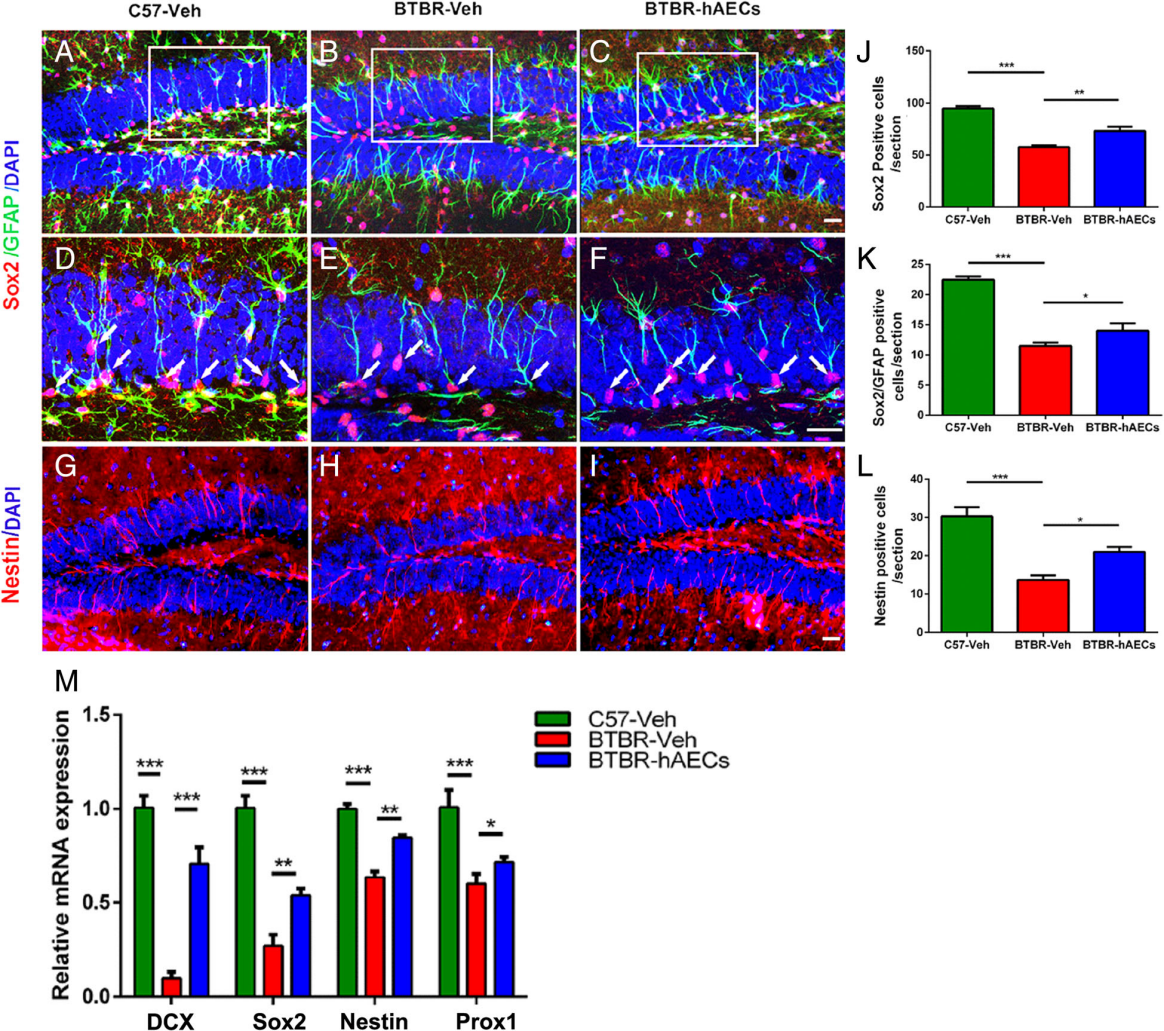
hAEC treatment enriched the NPC pool in the DGs and showed molecular beneficial effects in BTBR mice. **a**–**c** Representative images of Sox2 and GFAP double-positive RGCs in the DGs. **d**–**f** The images are the magnified views in **a**–**c**. The arrows indicate the Sox2 and GFAP double-stained cells. **g**–**i** Representative images of Nestin-positive RGCs in the DGs. **j** Quantitative analysis of the numbers of Sox2-positive cells in the DG-SGZ. of Vehicle-treated BTBR mice exhibited less Sox2-positive cells in the DG-SGZ compared to C57 mice, and the number of Sox2-positive cells was increased after hAEC treatment in BTBR mice. **k** Quantitative analysis of the number of Sox2^+^/GFAP^+^ cells in the DG-SGZ. vehicle-treated BTBR mice also exhibited less Sox2^+^/GFAP^+^ cells in the DG-SGZ compared to C57 mice, and the number of Sox2^+^/GFAP^+^ cells was increased after hAEC treatment in BTBR mice. **l** Quantitative analysis of the number of Nestin^+^ cells in the GCL. C57 mice exhibited more Nestin-positive cells in the GCL compared to vehicle-treated mice, and the number of Nestin-positive cells was increased after hAEC treatment in BTBR mice. The data are presented as the mean ± SEM (*n* = 4). **i** Relative mRNA expressions of DCX, Sox2, Nestin, and Prox1, which are expressed in low levels in BTBR mice treated with vehicle compared to C57 mice but increased after hAEC treatment in BTBR mice. *P < 0.05, **P < 0.01, ***P < 0.001. The scale bar in **c** = 50 μm and applies to **a**–**c**; in **f** = 20 μm and applies to **d**–**f**, and in **i** = 50 μm and applies to **g**–**i**

It is supposed that a decreased cellular proliferation in the adult SGZ may be induced by a reduction in NPCs. To assess the number of NPCs in the DG of BTBR mice, we compared the number of Sox2-labeled NPCs among the groups. BTBR mice treated with vehicle had fewer Sox2-stained NPCs in the DG-SGZ than C57 mice (Fig. 5a, b, d, e, j, *P* < 0.001). However, hAECs markedly increased Sox2-stained NPCs in the DG-SGZ of BTBR mice (Fig. 5b, c, e, f, j, *P* < 0.05). NPCs are mainly derived from RGCs in the DG-SGZ of the adult hippocampus, which are usually double stained with GFAP and Sox2 and are characterized by their vertical radial processes projecting across the GCL. BTBR mice with vehicle treatment have a smaller number of GFAP^+^/Sox2^+^ double-stained NPCs in the DG-SGZ than C57 mice (Fig. 5a, b, d, e, k, *P* < 0.001). hAECs markedly increased the number of GFAP^+^/Sox2^+^ double-stained RGCs in the DG-SGZ of BTBR mice by 21.7% (Fig. 5b, c, e, f, k, *P* < 0.05). What is more, BTBR mice treated with vehicle had fewer Nestin-stained NPCs in the DG than C57 mice (Fig. 5g, h, l, *P* < 0.001), while hAECs increased the number of Nestin-stained NPCs in the DG of BTBR mice (Fig. 5h, i, l, *P* < 0.05). Altogether, it indicates that hAECs promote maintenance of the NSC population.

Nestin and Sox2 are markers of stem/early progenitor cells, and real-time PCR results indicated that the levels of Nestin (Fig. 5m, *P* < 0.001) and Sox2 (Fig. 5m, *P* < 0.001) in the vehicle-treated BTBR mice were lower compared to that in the C57 mice. Moreover, Nestin (Fig. 5m, *P* < 0.01) and Sox2 (Fig. 5m, *P* < 0.05) were both upregulated in hAEC-treated BTBR mice. Similar to DCX and Prox1 staining, real-time PCR results showed that vehicle-treated BTBR mice showed a lower level of DCX (Fig. 5m, *P* < 0.001) and Prox1(Fig. 5m, *P* < 0.001) than the C57 mice; hAEC treatment increased DCX (Fig. 5m, *P* < 0.001) and Prox1(Fig. 5m, *P* < 0.05) expression in the hippocampus in BTBR mice.

### hAEC injection enhanced BDNF-TrkB signaling pathway in the hippocampus of BTBR mice

**Fig. 6:**
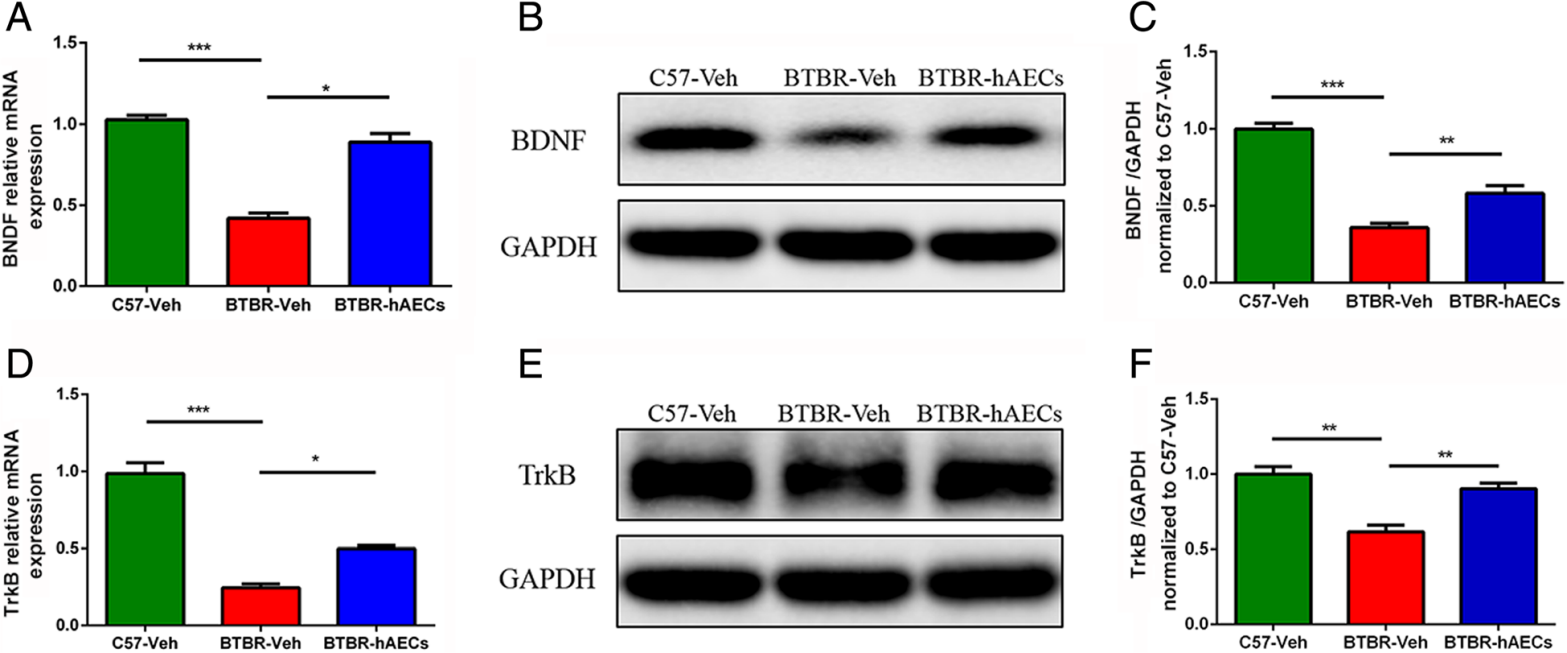
hAEC treatment increased the levels of BDNF and TrkB in the hippocampus of BTBR mice. **a** Relative mRNA levels of BDNF from the hippocampus in each group. **b** Representative western blotting for the BDNF protein from the hippocampus in each group. **c** Densitometric quantification of BDNF. **d** Relative mRNA levels of TrkB from the hippocampus in each group. **e** Representative western blotting for the TrkB protein from the hippocampus in each group. **f** Densitometric quantification of TrkB. Data are presented as mean ± SEM (*n* = 3); *P < 0.05, **P < 0.01, ***P < 0.001

BDNF and its receptor TrkB play key roles in the enhancement of neurogenesis. Real-time PCR results indicated that BTBR mice treated with vehicle showed lower mRNA levels of BDNF (Fig. 6a, *P* < 0.001) and TrkB (Fig. 6d, *P* < 0.001) compared to C57 mice. Moreover, BDNF mRNA (Fig. 6a, *P* < 0.05) and TrkB mRNA (Fig. 6d, *P* < 0.05) in the hippocampus were both upregulated in hAEC-treated BTBR mice. Consistent with mRNA expression, western blot showed that the protein levels of BDNF (Fig. 6b, c, *P* < 0.01) and TrkB (Fig. 6e, f, *P* < 0.01) in the hippocampus of BTBR mice were lower than those in the C57 mice, which could be increased by hAEC treatment. It is possible that the increase in BDNF and TrkB is responsible for the effects of hAECs on the promotion of hippocampal neurogenesis described here.

### Detection of hAECs 4 weeks after transplantation

**Fig. 7:**
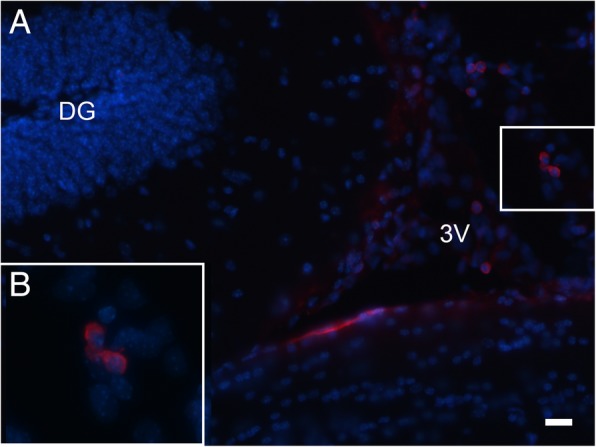
Identification of hAECs in the brain of mice 4 weeks after intracerebroventricular transplantation. (A) Identification of hAECs in the brain of mice 4 weeks after intracerebroventricular transplantation. Anti-human nuclei-positive cells (red) counterstained for DAPI nucleic acid staining (blue) identified adjacent to the wall of the dorsal third ventricle of a transplanted BTBR mouse. (B) Magnified views of hAECs. The scale bar in (A) = 20 μm

Four weeks after hAEC transplantation, human nuclei (HuNu)-positive cells were mainly found within the walls of the dorsal third ventricle and lateral ventricle of the BTBR mice (Fig. 7). Few positive cells were detected in other brain regions.

## Discussion

Recently, hAECs are considered as ideal stem cell candidate for regenerative medicine. Many studies have confirmed the therapeutic effect of hAECs in various tissues, including the brain [35–37]. The present study described the behavioral and molecular beneficial effects of hAEC transplantation in BTBR mice, an ASD animal model. We found that a single hAEC transplantation in young adult BTBR mice caused a significant improvement in sociability. We further confirmed that enhanced hippocampal neurogenesis is involved in the rescue of social deficits, which are partly due to the modulation of BDNF and TrkB within the hippocampus.

To investigate the effects of hAEC transplantation on treating the core symptoms of ASD, we employed the BTBR inbred mouse strain, which exhibits social abnormalities and repetitive behaviors [21]. In three-chambered social approach task, BTBR mice receiving hAEC transplantation displayed a strong preference for the novel mouse over the object as detected by the time spent in the chamber and sniffing. Considering the social behaviors in mice could be affected by the exploratory activity and motor abilities, we further detected the effects of hAEC transplantation on exploratory locomotion. A standard 30-min open-field test was used to determine the general exploratory locomotion of mice. The total distance traveled by BTBR mice was not altered by hAEC transplantation, indicating that the restored social behaviors by hAEC transplantation were typical in BTBR mice. The self-grooming and marble burying tests represent repetitive behavior tests, and we noticed that hAEC transplantation did not improve any of the repetitive behaviors. Considering the complexity of neurodevelopment, the rapidity of early neurogenesis, and the increased prevalence of ASD, it is possible to postulate that stem cell therapy may be varied based on the time point for treatment, which shows different efficacies of therapeutic effects depending upon the severity of the disease. Thus, it may be required to perform comprehensive time response curves to determine the optimal treatment regimen. Moreover, since BTBR mice are a multifactorial model of autism, it is not clear what is the exact genetic aberration in this strain that is responsible for the autism-like phenotype. It may be worth to test the effect of hAEC transplantation in other genetically modified mice.

Recently, more studies have indicated that neurogenesis is a critical cellular process involved in ASD pathophysiology [38, 39]. We have previously confirmed that the loss of LXRβ-induced ASD-like behaviors is closely related to inhibited hippocampal neurogenesis [8]. Clinical studies have suggested that ASD patients display defects in neurogenesis. In this study, hAEC transplantation rescued the inhibition of hippocampal neurogenesis significantly in BTBR mice, as determined by increased BrdU-positive cells, DCX-positive neurons, and Prox1-positive neurons in the hippocampal DG. RGCs in the adult hippocampus produce NPCs and contribute to the maintenance of the NPC pool, which determines the capacity of adult hippocampal neurogenesis [40]. Our results demonstrated that hAEC transplantation into young adult BTBR mice increased RGCs in the DG, which may lead to the recovered neurogenesis in BTBR mice.

As it is widely accepted, grafted stem cells in the CNS regenerates or repairs the nervous system through several mechanisms including neurotrophic support, anti-inflammatory effects, and cell replacement [41]. hAECs were proved to differentiate into cortical progenitors in vitro [42] and induced dopaminergic neuron-like cells differentiation of human umbilical cord blood-derived mesenchymal [43]. However, there are no evidences showing that grafted hAECs differentiated into neurons in the CNS. In this study, we found the survived hAECs were identified adjacent to the walls of the dorsal third ventricle and lateral ventricle of the BTBR mice after 4 weeks post-transplantation of hAECs; hAEC-derived neurons were not observed in the hippocampus and other brain regions. It might be inferred that cell replacement is not the main mechanism in the correction of hippocampal neurogenesis and behavioral deficits.

It is generally accepted that hAECs maintain an earlier embryologic phase, are much younger, and could act as “biological minipumps” within the central nervous system, secreting a wide range of multifunctional factors. It has been confirmed in several studies that the factors secreted by hAECs promote the survival of neurons and enhance the neurogenesis. These paracrine factors released from hAECs included pleiotrophin [44], proangiogenic cytokines [45], and neurotrophins such as BDNF and NT-3 [46, 47]. Interestingly, our results show that hAEC transplantation increased the levels of BDNF and TrkB in the hippocampus of BTBR mice. We infer that hAEC-induced increases in BDNF levels may underpin the enhanced neurogenesis in hAEC-treated BTBR mice, thus resulting in behavioral improvement.

Neuroinflammation has been detected in the hippocampus of BTBR mice, which may influence adult hippocampal neurogenesis. It has been indicated that hAECs play beneficial roles by preventing inflammatory responses [48–50]. Evidence has confirmed that hAECs have the potential to treat autoimmune diseases via inhibiting the activity of immune cells and could improve stroke outcome by suppressing pro-inflammatory cytokines and secreting factors that inhibit the chemotactic activity of neutrophils and macrophages [20]. Here, we observed that neither microglial activation nor inflammatory factors were altered by hAEC transplantation. This finding suggests that the immunomodulatory mechanism was not involved in the recovery of hippocampal neurogenesis in the BTBR mice followed by hAEC transplantation.

## Conclusions

The findings in this study first showed the potential of hAEC transplantation in an ASD mouse model and presented new data deciphering the possible mechanisms in which hAEC transplantation may result in structural brain changes that could rescue behavioral deficits. Specifically, we found that hAEC transplantation increased BDNF and TrkB expression and was related to enhanced hippocampal neurogenesis. Future studies that investigate the underlying mechanisms in animal models of ASD may provide a better understanding of ASD pathogenesis and pave the way for hAEC treatment in ASD patients.

## Data Availability

All data generated or analyzed for this study are included in this published article and the additional files.

## Abbreviations

ASD: Autism spectrum disorder
BDNF: Brain-derived neurotrophic factor
BTBR: BTBR T+tf/J
DG: Dentate gyrus
GCL: Granule cell layer
HAECs: Human amniotic epithelial cells
MSC: Mesenchymal stem cell
NPCs: Neural progenitor cells
PFA: Paraformaldehyde
RGCs: Radial glial cells
SGZ: Subgranular zone
VPA: Valproic acid
